# High-throughput automated molecular replacement for small-molecule MicroED data

**DOI:** 10.1107/S2052252526002095

**Published:** 2026-04-10

**Authors:** Adam Thibodeaux, Guanhong Bu, Lael C. Edwards, Emma Rova Danelius

**Affiliations:** a Department of Chemistry University of California, Riverside 900 University Avenue Riverside CA 92521 USA; Ben-Gurion University of the Negev, Israel

**Keywords:** small-molecule electron diffraction, MicroED, 3DED, electron crystallography, molecular replacement, high-throughput methods, automation algorithms

## Abstract

Interest in electron diffraction (ED) for structural characterization of both proteins and small molecules has grown significantly over the last decade. While *ab initio* phasing methods remain the gold standard for ED data from small-molecule samples, radiation beam damage during data collection and poor crystallinity of the nanocrystalline sample can make this method unfeasible – particularly for challenging molecules that exhibit conformational flexibility. Molecular replacement (MR) is the most commonly used phasing method for protein ED data and can circumnavigate issues related to diminished data quality. However, its application to small molecules has been limited due to the lack of methods for generating optimal trial conformations. Herein, a high-throughput automated molecular replacement workflow has been developed to solve a novel ED structure of corilagin, a macrocyclic gallotannin with pharmaceutical relevance, which could not be solved with *ab initio* phasing. The method was validated against three similar macrocycles with known structures (paritaprevir-α, paritaprevir-β and grazoprevir) at varying data resolution limits (1.0, 1.2, 1.4, 1.5, 1.6, 1.8 and 2.0 Å). At all these resolutions for all three structures, the developed workflow was successful and produced solutions with *R* factors and RMSD values within acceptable limits of the *ab initio* solved structure.

## Abstract 2

A new algorithm based on molecular replacement has been developed for structure determination of small molecules from MicroED data with resolutions from 0.85 to 2.0 Å.

## 1. Introduction

Microcrystal electron diffraction (MicroED), also known as 3D electron diffraction (3DED), has seen a steady gain in traction in the field of crystallography over the last decade (Shi* et al.*, 2013 ▸; Jones *et al.*, 2018 ▸; Mu *et al.*, 2021 ▸; Gemmi *et al.*, 2019 ▸; Danelius *et al.*, 2023*b* ▸; Haymaker & Nannenga, 2024 ▸). For small-molecule structures, following the early developments of *ab initio* determination directly from powder formulations (Gorelik *et al.*, 2012 ▸; van Genderen *et al.*, 2016 ▸; Jiang *et al.*, 2022 ▸), recent advances have demonstrated the ability to determine hydrogen-atom positions (Palatinus *et al.*, 2017 ▸) and absolute configuration (Brázda *et al.*, 2019 ▸), highlighting the method’s potential and applicability. The ease of sample preparation represents a significant advantage of MicroED for small molecules and has contributed substantially to its widespread adoption as a structure determination technique. In contrast, sample preparation remains a challenging and rate-limiting step in macromolecular MicroED. For small-molecule analyses, nanocrystalline material is frequently obtainable directly from powdered samples, thereby eliminating the need for crystallization screening. Nevertheless, powder-derived specimens may still present obstacles to achieving high-resolution diffraction data. Such limitations typically result from inadequate crystallinity or from electron-beam-induced radiation damage during data acquisition (Hattne, 2021 ▸). These limitations render a significant number of datasets from MicroED experiments (∼50% of datasets from complex molecules, from our experience) impossible to phase by direct methods, *i.e.* solving the phase problem based on the diffraction amplitudes alone (Sheldrick *et al.*, 2012 ▸). In situations where the data resolution is not sufficient for direct methods, global optimization techniques, such as simulated annealing, have successfully been used for crystal structure determination from ED data (Feyand *et al.*, 2012 ▸; Burla *et al.*, 2015 ▸; Gemmi *et al.*, 2019 ▸; Woollam *et al.*, 2020 ▸; Andrusenko *et al.*, 2020 ▸; Lightowler *et al.*, 2022 ▸). Global optimization utilizes knowledge about the connectivity of the molecule (Brunger, 1991 ▸; Burla *et al.*, 2015 ▸; Woollam *et al.*, 2020 ▸), a condition that holds in many cases.

In the realm of protein crystallography, where poorer quality diffraction data are the *de facto* norm due to similar poor crystallinity (Timofeev & Samygina, 2023 ▸), molecular replace­ment (MR) is the most effective and widely used alternative to *ab initio* methods, representing more than 80% of X-ray structures deposited in the Protein Data Bank (PDB) (Burley & Berman, 2021 ▸). MR is a conceptually simple method where the position and orientation of a trial structure in the crystal is optimized using a series of translational and rotational functions to best align the calculated structure-factor amplitudes from the structure in a particular position in the crystal lattice with structure factors determined from the experimental diffraction data (Rossmann, 1990 ▸). A major fragment of the trial structure needs to share the same conformation as the correct structure for successful MR. If the fragment is unknown, conformational searches using global optimization methods such as simulated annealing will be essential for screening different conformations. Because the basis for the optimization in an MR algorithm relies heavily on differences in structure factors between the calculated and experimental data, it is readily apparent that the conformation of the trial structure input into the MR algorithm is of critical importance. MR will only succeed when the trial model closely resembles the true structure (Abergel, 2013 ▸).

With the recent surge in popularity of large, complex and conformationally flexible molecules as drug candidates that can target previously undruggable proteins [*e.g.* flat or featureless proteins with no apparent binding site (DeGoey *et al.*, 2018 ▸)], structural elucidation of these compounds is important for further development. This class of compounds are referred to as ‘beyond Rule of 5’ (bRo5) compounds, as their molecular properties (molecular weight, lipophilicity, number of hydrogen-bond donors and acceptors) are beyond the Lipinski Rule of 5 (Ro5) (Lipinski *et al.*, 1997 ▸). bRo5 compounds, such as macrocycles, are known to display inherent flexibility and are best described as conformational ensembles in solution. Because bond rotations in macrocycles are highly constrained and correlated, generating the full conformational space is computationally demanding; as a result, global optimization methods are rarely used as the primary approach for producing macrocyclic conformational ensembles. Instead, many conformer generators exist based on a myriad of methods [*e.g.* machine learning (Jiang *et al.*, 2022 ▸), molecular mechanics (Watts *et al.*, 2014 ▸), molecular dynamics (Poongavanam *et al.*, 2018 ▸) and distance geometry (Seidel *et al.*, 2023 ▸)]. Recent work has shown that distance geometry methods are able to sample the conformational space to produce structures for these complex molecules within 2 Å RMSD cutoff of the experimentally determined structure, with noticeably improved efficiency compared with the more conventional molecular mechanics methods (Seidel *et al.*, 2023 ▸). While an RMSD of 2 Å is within the suggested limit for MR in protein crystallography, the RMSD cutoff of 0.25 Å for MR of small molecules is much lower (van de Streek & Neumann, 2010 ▸). Some recent work has shown that MR can indeed be applied to small-molecule systems when combining this phasing method with fragmentation of the target molecule into rigid fragments and conformation screening using a multicomponent MR search (Gorelik *et al.*, 2023 ▸; Deschner *et al.*, 2025 ▸). This approach will prove useful for traditional Ro5 compounds that can be easily fragmented into rigid moieties, but is not easily amenable to the bRo5 candidates that lack any obvious fragmenting patterns.

**Figure 1:**
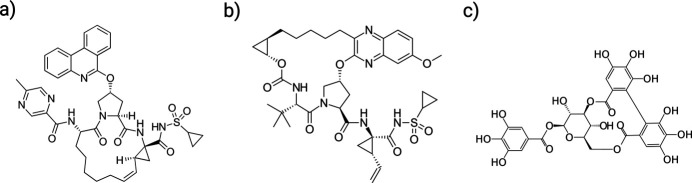
Macrocyclic ‘beyond Rule of 5’ (bRo5) molecules used to validate the HAMR method: (*a*) paritaprevir, (*b*) grazoprevir and (*c*) corilagin.

**Figure 2:**
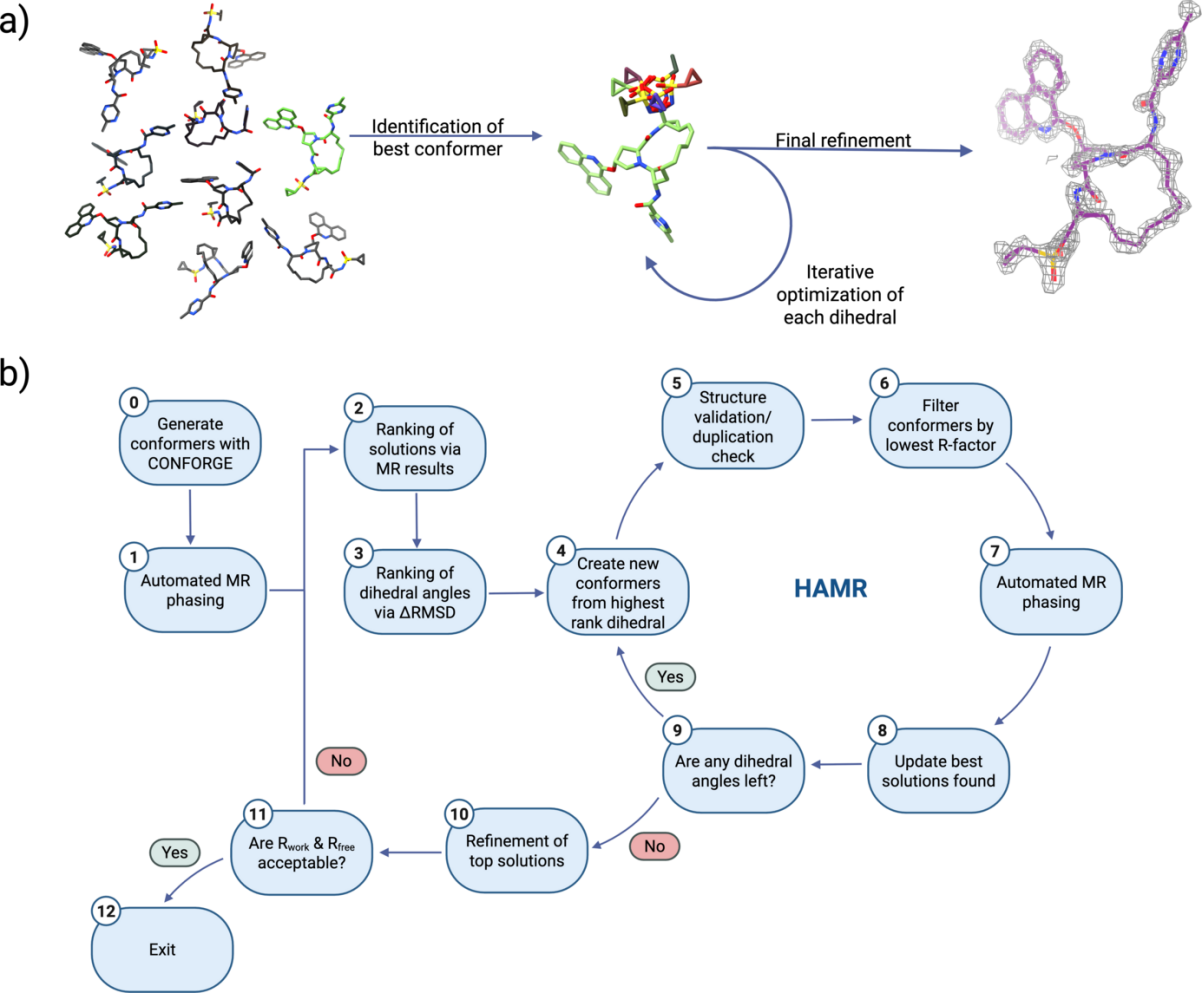
(*a*) Visual representation of HAMR algorithm logic as applied to a validation compound – paritaprevir-α at 1.4 Å data resolution. (*b*) Generalized logical flowchart for the most significant steps involved in the HAMR algorithm. In step 2, solutions can be ranked via LLG or *R* factor. In step 3, ΔRMSD represents the average RMSD change for each dihedral angle during a full 360° rotation in steps of 10°. In step 6, the amount of filtering is specified during setup and can be omitted for a more complete search of conformational space.

Herein, we expand the capabilities of MR applied to small-molecule systems by designing an algorithm to automate the process of solving the structure for macrocyclic bRo5 molecules using MicroED data without any fragmentation. The input ensemble for our automated MR was calculated using the open-source conformer ensemble generation tool *CONFORGE* (Seidel *et al.*, 2023 ▸). For sampling macrocycle conformers, this tool implements a purely stochastic approach based on distance geometry and force-field-driven structure refinement. Paritaprevir and grazoprevir [Fig. 1 ▸(*a*) and (*b*)] were chosen as validation structures to ensure that the high-throughput automated molecular replacement (HAMR, Fig. 2 ▸) algorithm works properly, and corilagin [Fig. 1 ▸(*c*)] was chosen as a novel structure to highlight the capability of the algorithm.

Paritaprevir and grazoprevir are both exemplary validation targets, as they are early bRo5 compounds with successful use in the clinic, both being FDA approved macrocyclic drugs, and both having MicroED structures solved using *ab initio* methods (Danelius *et al.*, 2023*a* ▸; Bu *et al.*, 2024 ▸; Wieske *et al.*, 2026 ▸). Additionally, paritaprevir is an especially good candidate for validation as there are two known polymorphic conformers (referred to here as paritaprevir-α and paritaprevir-β), with an all-atom RMSD of approximately 0.8 Å, and it is imperative that the developed method can correctly discern between these to be useful for MicroED data that may represent multiple conformers. Finally, corilagin was chosen both due to its multiple potential medical uses documented in the literature (Li *et al.*, 2018 ▸), and as a real scenario of a MicroED dataset that was not possible to solve with *ab initio* methods despite a plethora of good quality data.

## 2. Materials and methods

### 2.1. Materials

Corilagin was commercially available from Invivochem. Approximately 0.5 mg powder was dissolved in ultrapure H_2_O in a 4 ml scintillation vial and air dried in a fume hood. The dried residue was scraped from the glass wall.

### 2.2. Grid preparation

The grid preparation followed the procedure as described previously (Unge *et al.*, 2024 ▸; Lin *et al.*, 2024 ▸). One TEM grid coated with continuous carbon support film (200 mesh, 3.05 mm OD, Ted Pella) was prepared by glow discharging in the negative mode for 60 s on each side at 15 mA in a PELCO easiGlow (Ted Pella). The grid was gently mixed with the scraped dry residue in the scintillation vial, taken out and clipped for loading into a cryo-TEM.

### 2.3. MicroED data collection

The MicroED data collection followed the procedure described previously (Unge *et al.*, 2024 ▸; Lin *et al.*, 2024 ▸), using *EPU-D* (Thermo Fisher Scientific) on a Talos Arctica cryogenic transmission electron microscope (Thermo Fisher Scientific) operating at 80 K with an acceleration voltage of 200 kV, corresponding to a wavelength of 0.0251 Å. The whole-grid atlas was acquired at a magnification of 210×. Microcrystals were screened at a magnification of 3400×. Upon identification of single microcrystals, the selected area aperture size 50 (1.4 µm in diameter) was inserted, and the microscope was switched to diffraction mode for taking still diffraction images under the eucentric height and parallel electron beam conditions (C2 lens intensity 45.2%, C2 aperture size 70 and spot size 11). Once high-resolution diffraction spots were observed, MicroED data were continuously recorded on the Falcon III detector (Thermo Fisher Scientific) at an exposure rate of 1 second per frame as the stage was continuously rotating from −69° to +69° at a speed of 0.6° per second. Each single-crystal dataset was collected as a movie in MRC format at a total electron fluence of 2.30 e Å^−2^.

### 2.4. MicroED data processing

For each single-crystal dataset, the diffraction images were extracted from the raw MRC movie and converted to SMV format using the *MRC2SMV* software freely available at https://cryoem.ucla.edu/microed (Hattne *et al.*, 2015 ▸). The diffraction images were processed in *XDS*, in which a resolution cutoff was applied at a mean *I*/σ(*I*) ≥ 1.0 and CC_1/2_ ≥ 0.3 in the highest resolution shell (Kabsch, 2010 ▸; Brehm *et al.*, 2023 ▸). Reflection intensities from different single-crystal datasets were scaled and merged in *XSCALE* (Kabsch, 2010 ▸; Brehm *et al.*, 2023 ▸). Reflections were converted to *SHELX* HKL format in *XDSCONV* (Kabsch, 2010 ▸; Brehm *et al.*, 2023 ▸). For corilagin, since no solution could be obtained from *ab initio* phasing using *SHELXT* or *SHELXD* (Sheldrick, 2015 ▸; Schneider & Sheldrick, 2002 ▸), the reflections were converted to *CCP4* MTZ format in *XDSCONV* (Kabsch, 2010 ▸; Brehm *et al.*, 2023 ▸) for phasing using MR. For grazoprevir, paritaprevir-α and paritaprevir-β, the reflection files from previously published MicroED structures (Danelius *et al.*, 2023*a* ▸; Bu *et al.*, 2024 ▸; Wieske *et al.*, 2026 ▸) were directly used and converted from *SHELX* HKL format to *CCP4* MTZ format using *F2MTZ* in the *CCP4i* program suite (Potterton *et al.*, 2003 ▸). Resolutions were cut to 1.0, 1.2, 1.4, 1.5, 1.6, 1.8, 2.0, 2.5 and 3.0 Å using the highest resolution *CPP4* MTZ reflection files with *mtzutils* in the *CCP4* program suite to simulate low-resolution data for paritaprevir-α, paritaprevir-β and grazoprevir (Potterton *et al.*, 2003 ▸). The crystallographic data for corilagin can be found in Table 2, and the data for grazoprevir, paritaprevir-α and paritaprevir-β in the supporting information, Tables S3–S25.

### 2.5. Automated molecular replacement

Initial conformers were generated from SMILES notation using the *CONFORGE* package as provided within the Python bindings for the C++ CDPKit library, with default settings including 20 kcal mol^−1^ energy window, 300 output conformers, 0.5 Å minimum RMSD between conformers and 2 h maximum generation time (Seidel *et al.*, 2023 ▸). Additionally, the number of sampled conformers was unlimited for *CONFORGE* conformer generation for corilagin, which was necessary to sample a wider range of the conformational landscape of this molecule. Similar settings were used for conformer generation using RDKit’s *EKTDGV3* (Wang *et al.*, 2020 ▸).

During HAMR cycles, conformers were generated by modifying dihedral angles using Python bindings for the C++ RDKit library. Structure factors were calculated using Python bindings for the C++ gemmi library via the provided electron density calculator in electron scattering mode with approximate isotropic temperature factor fitting and scaling without any solvent mask (Wojdyr, 2022 ▸). Molecular replacement was performed using the *PHASER* method as provided in the *CCP4* program suite by utilizing the binary executable directly (Potterton *et al.*, 2003 ▸; McCoy *et al.*, 2007 ▸).

As a part of the pre-validation check before initiating MR in *PHASER*, the algorithm performs a composition check to ensure the volume of the structure will fit inside the asymmetric unit (ASU) defined by the unit-cell dimensions (McCoy *et al.*, 2007 ▸). However, because *PHASER* was developed almost exclusively for proteins and other macromolecules, with only a small nod to small molecules as protein ligands, this composition check is often inaccurate for small molecules, resulting in an incorrectly failed pre-validation check. To circumvent this, a workaround was used where the composition of the unit cell was defined only as a lone hydrogen atom, which will always pass this pre-validation check. This composition is later updated during the *PHASER* algorithm with the actual provided structure after this pre-validation is complete and successful, thereby not reducing the validity of any of the results of MR.

### 2.6. Refinement

Refinement was performed on solutions after completion of HAMR cycling using the *refine* program as provided in the *PHENIX* program suite by utilizing the binary executable directly (Liebschner *et al.*, 2019 ▸). The *R*_free_ test set was generated before every refinement at 5% of the total intensities, except for paritaprevir-α at 1.8 Å resolution, where 10% of the total intensities were used. Individual sites in reciprocal space, individual sites in real space, individual isotropic atomic displacement parameters, simulated annealing in Cartesian and torsion space, optimization of XYZ weighting, and optimization of ADP weighting using the electron scattering form factors were performed for five cycles each for all refinements.

## 3. Results

### 3.1. Initial fragmentation MR of known structures

Initially, we attempted to utilize the fragmentation-based multicomponent search MR for small molecules developed by Gorelik *et al.* (Gorelik *et al.*, 2023 ▸). However, as is readily apparent in the structures for both paritaprevir and grazoprevir shown in Fig. 1 ▸, both lack any obvious way to fragment the compound into solely rigid fragments. While paritaprevir does contain the pyrimidine and benzo­quinoline side chains which are rigid, the sulfonyl side chain and core have significant flexibility. Similarly, grazoprevir contains a *tert*-butyl side chain, which is rigid, but again the sulfonyl side chain and core have significant flexibility. Despite this, a fragmentation strategy was used as an initial trial set where the macrocyclic core and all side chains, regardless of flexibility, were separated into individual fragments as our best approximation of the appropriate MR search input structures. Attempting this in all possible permutations of fragments and core unfortunately did not result in a solved structure, highlighting the necessity of rigid moieties when using the fragmentation-based MR procedure.

### 3.2. Initial conformer generation analysis

We devised a new strategy wherein a series of conformers were generated for each compound and then individually phased using MR in an automated fashion using a Python script. We tested two open-source, freely available conformer generators based on distance geometry (*CONFORGE* and *EKTDGV3*) for this conformer generation. The initial MR phasing results were evaluated by the log-likelihood gain (LLG) and the translation function *Z*-score (TFZ), the two best predictors of model accuracy when using MR (McCoy *et al.*, 2005 ▸). Although it has been suggested that for small molecules TFZ- and LLG-based data analysis cannot readily discriminate structures that are somewhat similar to the correct structure (Gorelik *et al.*, 2023 ▸), these metrics do serve as a useful tool for eliminating faulty solutions that are not at all similar to the correct solution. Our analysis showed that of the two conformer generators, the average LLG and TFZ score for grazoprevir, paritaprevir-α and paritaprevir-β are best for *CONFORGE*, suggesting that this conformer generator produces the highest quality conformers for our method (Table S1).

Although this trend is useful and did point us in the right direction, we were unable to arrive at any solution with acceptable *R*_work_, *R*_free_ or a qualitatively low RMSD compared with the solution found using *ab initio* methods after refinement, and further modification of the conformers was necessary to successfully produce a valid solution via MR.

### 3.3. HAMR of grazoprevir, paritaprevir-α and paritaprevir-β

A scheme was devised that involved the automated modification of the conformers generated by *CONFORGE* by modifying each dihedral angle individually and determining the accuracy of these modified conformers using both the *R* factor of the initially phased electrostatic potential map and the LLG from the re-phased modified conformer, which is shown visually in Fig. 2 ▸. In an automated fashion, this process was repeated for each dihedral angle that is not within the macrocycle core – which is avoided due to the complexity of modifying dihedral angles within a cyclic structure while maintaining the correct chemical bonding. Instead, sampling of conformational space of the macrocyclic core is handled by *CONFORGE*, as for each HAMR attempt a different input structure with a different core conformation is optimized. This system was designed as a genetic algorithm, where the top solutions from each cycle are used as starting models for the next cycle until all dihedral angles have been fully optimized, as this problem can be viewed as a simple global optimization problem of LLG, which is well suited for genetic algorithms (Katoch *et al.*, 2021 ▸).

After selection of the best *CONFORGE* conformer and modification of the selected dihedral angle, the *R* factor of the modified conformer in the same position in the crystal lattice as the previously phased result is used as a form of filtering to determine which modified conformers are worth performing MR on. This was done as the MR process is the largest bottleneck in this algorithm, and from our testing this *R* factor serves as an approximate proxy for determining the initial validity of the modified conformers, despite still needing MR for determination of the pose of the conformer within the crystal lattice to arrive at a final solved structure. Similarly, to avoid running unnecessary MR computations, a de-duplication check is performed each cycle, wherein conformers within 0.02 Å RMSD of an already phased solution are discarded, as increased structural diversity of conformers tested represents a wider search of total conformational space.

**Figure 3:**
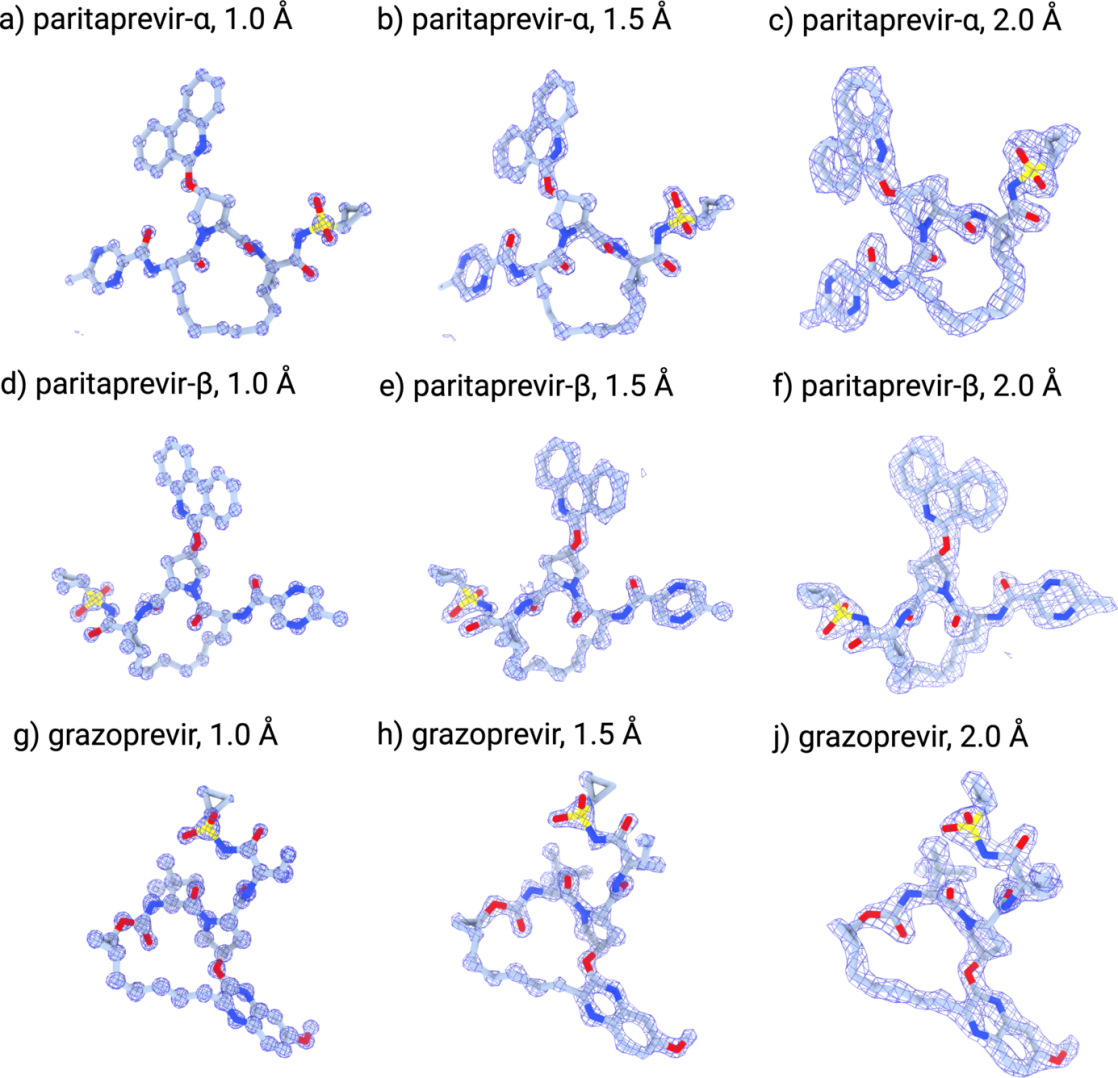
HAMR structure solutions (light blue sticks) for paritaprevir-α (*a*–*c*), paritaprevir-β (*d*–*f*) and grazoprevir (*g*–*j*) at varying resolutions with the resultant 2*mF*_o_ − *DF*_c_ map (dark blue mesh). 2*mF*_o_ − *DF*_c_ maps are contoured at 3.0, 2.5 and 1.5σ above the mean for 1.0, 1.5 and 2.0 Å resolution cutoffs, respectively.

As previously mentioned, this method was applied to grazoprevir, paritaprevir-α and paritaprevir-β at each dataset’s highest resolution (0.99, 0.85 and 0.95 Å, respectively) along with 1.0, 1.2, 1.4, 1.5, 1.6, 1.8, 2.0 and 2.5 Å resolution as a simulation of lower quality data, for which all results are summarized in Table 1 ▸; further refinement statistics are provided in Tables S3–S27. Example electrostatic potential maps for all validation compounds are included in Fig. 3 ▸, showing that all atoms fall within the electrostatic potential map, but the sharpness of the map dramatically decreases with decreasing resolution. We investigated the observed trends in RMSD, *R*_work_ and *R*_free_ by performing MR and refinement with the same settings on the *ab initio* solved structure and observed similar trends compared to the HAMR solved structures (Table S2).

### 3.4. HAMR of corilagin

**Figure 4:**
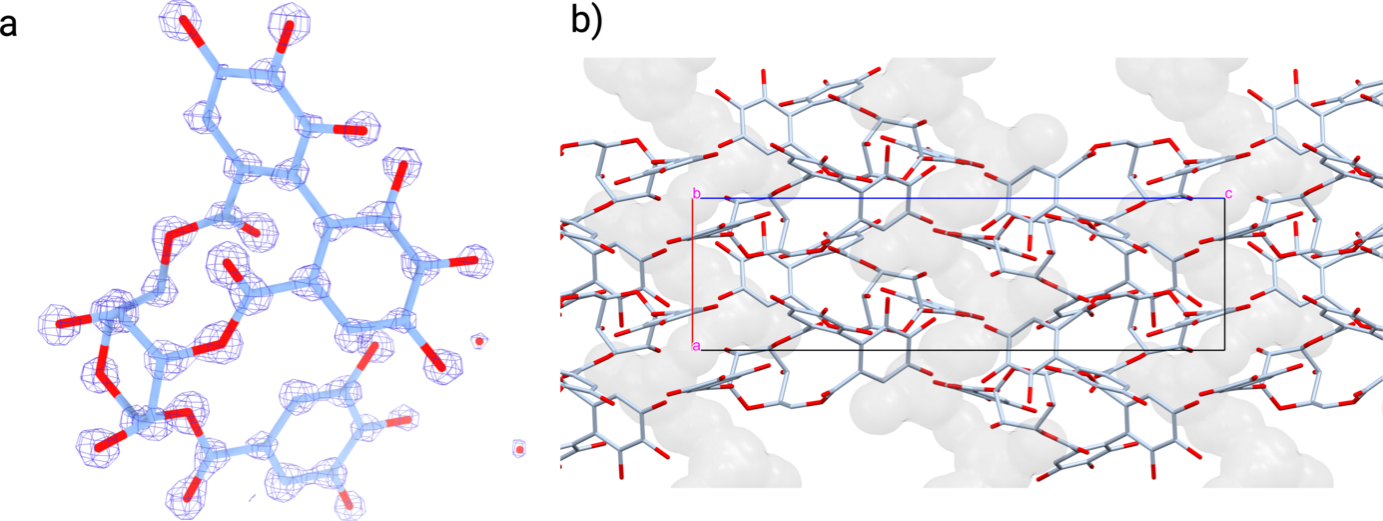
(*a*) HAMR solution structure (light blue sticks) of corilagin at 1.1 Å resolution with resultant 2*mF*_o_ − *DF*_c_ map (dark blue mesh). The 2*mF*_o_ − *DF*_c_ map is contoured to 3.0σ above the mean. (*b*) Crystal packing analysis of HAMR output for the corilagin structure shows void volumes along the crystallographic *a* axis.

After we had validated the developed method on grazoprevir, paritaprevir-α and paritaprevir-β, we set out to productively use this method by solving the structure of another macrocycle without a known structure, corilagin, which our lab has been unable to solve with *ab initio* methods, likely due to the disorder from the hexose ring and multiple hydroxyl groups (Willart *et al.*, 2010 ▸). The process for our HAMR method applied to corilagin was exactly the same as for the validation compounds except for including one additional setting in the initial *CONFORGE* conformer generation. In *CONFORGE*, the number of sampled conformers, which is normally limited to 2000 by default without issue, was set to unlimited. This molecule is somewhat less flexible than the validation compounds, resulting in an early exit of *CONFORGE* without sufficient sampling of conformational space due to the appearance of many duplicate conformers. The HAMR output structure showed void volumes along the crystallographic *a* axis [Fig. 4 ▸(*b*)]. Although voids do not inherently prevent structure determination, they can contribute to difficulties in solving the structure using *ab initio* methods, due to relatively poor and low-efficiency crystal packing (Steed & Steed, 2015 ▸). Additionally, unlike our validation compounds, weak positive electrostatic potential difference densities were observed in the HAMR output, which were attributed to two unmodelled water-molecule sites that were partially occupied in the void volumes, as the preparation of corilagin involved recrystallization from water. Currently HAMR does not support automatic addition of solvent molecules, so these water molecules were manually added to the crystal structure and refined once again with the same settings as used previously to arrive at the final structure. The data for the solved structure obtained from this HAMR run after manual solvent addition for corilagin are summarized in Table 2 ▸, and the structure is depicted in Fig. 4 ▸(*a*).

## 4. Discussion

### 4.1. Trends in data

**Figure 5:**
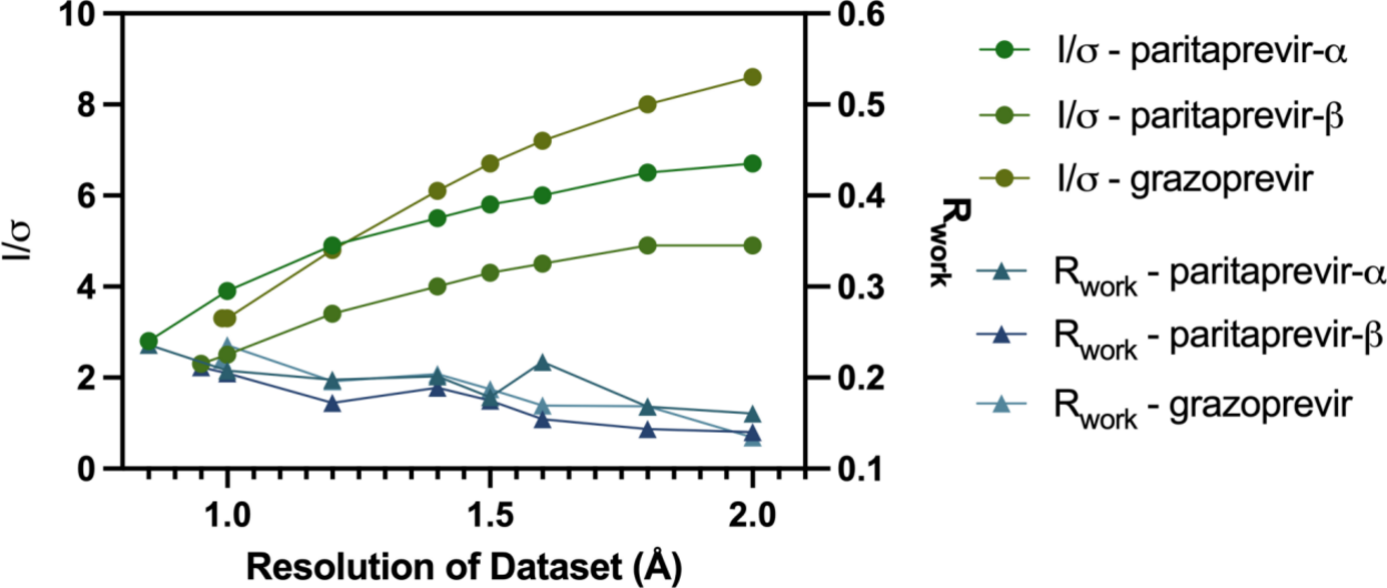
Comparison of refined *R*_work_ values for HAMR solutions for validation compounds to *I*/(*I*)σ values of the datasets with resolution cutoffs applied, displaying an inverse relationship between these two statistics.

As can be seen in Table 1 ▸, the *R* factors generally improve inversely with data resolution – *i.e.* lower-resolution MicroED data produce improved statistics until an absolute limit is reached wherein only limited useful structural information is able to be resolved from the MicroED data, which is 2.5 Å for all validation compounds. This inverse trend is primarily due to a noticeable increase in *I*/σ(*I*) for lower-resolution shells, as shown in Fig. 5 ▸; because the *R* factor is calculated as a function of the standard error in the intensities, it should rightfully decrease with a decreased standard error, which is what we observe. Furthermore, the RMSD between the *ab initio* solved structure and the HAMR solved structure has the opposite trend, generally increasing as a function of decreased resolution. Once again, this intuitively makes sense: as the resolution decreases, the number of reflections also decreases, leading to less structural information that can be determined from the reflections and a less accurate model at lower resolution. However, all RMSD values are reasonable and do not suggest that a different conformer is produced from HAMR compared with *ab initio* methods, as the RMSD values are well below the commonly used threshold for unique conformers of 0.5 Å RMSD (Olanders *et al.*, 2020 ▸). Instead, the RMSDs illustrate slight differences in atomic coordinates for every single atom, rather than drastically large conformational changes (Figs. S1–S3).

### 4.2. Limitations of HAMR

As mentioned previously, HAMR reliably solved the structures for paritaprevir and grazoprevir up to 2.0 Å. Beyond this resolution, however, the method failed to provide meaningful structural information. This threshold is evident from the *R* factors, which exceed 0.45 at 2.5 Å for all structures. Correspondingly, the RMSD values for our validation compounds also increase significantly – exceeding 1 Å in all cases – further indicating that the practical lower resolution limit is approximately 2 Å, as reflected in the observable *R* factors.

Additionally, at lower resolutions (>1.4 Å) for all compounds, the electrostatic potential map becomes less detailed, as can be seen in Fig. 3 ▸. This is expected, but does lead to indistinguishability of similar scatterers. This indistinguishability is a noticeable limitation at this resolution, as can be seen in Table 1 ▸, where the unadjusted RMSD for paritaprevir-β above 1.5 Å lies well above the RMSD values at higher resolutions. Because carbon and nitro­gen have somewhat similar scattering factors and also because of the blurring of the electron density, the method cannot easily distinguish between these atoms at the lower resolutions. Thus, the pyrimidine ring side chain on paritaprevir appears pseudo-*C*_2_ symmetric. This results in the output structure from HAMR methods above 1.4 Å for paritaprevir-β having no preference for this pyrimidine ring in the correct position or flipped 180° along the axis in the plane of the substituted ring. For this reason, we acknowledged this limitation and corrected this RMSD in Table 1 ▸ for paritaprevir-β, by calculating the RMSD based on atomic positions of heavy atoms alone not considering atomic species, which is shown in this table as the adjusted RMSD. After performing this correction, a similar trend to paritaprevir-α and grazoprevir – namely slightly decreasing the RMSD as a function of increasing resolution – is observed. However, at the high resolutions the method can easily distinguish between these atoms and arrives at the correct solution for both paritaprevir-α and paritaprevir-β.

Interestingly, we do not observe this same indistinguishability issue in paritaprevir-α, presumably due to the difference in data quality in these two datasets; while the completeness for both datasets are high, 89.0% and 98.4% respectively, paritaprevir-α has a noticeably higher signal-to-noise ratio than paritaprevir-β: the *I*/σ(*I*) values of the highest resolution dataset are 2.8 and 2.3, respectively. Thus, the improved *I*/σ(*I*) of the paritaprevir-α dataset contains enough information to effectively distinguish between carbon and nitro­gen in this case, suggesting that the HAMR method may be relatively robust to small differences in data completeness but is significantly sensitive to the noisiness of the data. We further observed that this correlation is also observed with the *R* factors – *i.e.* as *I*/σ(*I*) increases and the data become less noisy, both *R*_work_ and *R*_free_ improve, as can be seen in Fig. 5 ▸. This explains why the *R* factors, which are representative of model accuracy, improve with lower resolution despite the decreased amount of information present at lower resolutions, and further supports the observed sensitivity of our method to dataset noise.

Finally, we have only tested this method on pharmaceutically relevant macrocyclic small molecules. While in principle this method should work well for non-macrocyclic molecules, this remains unexplored territory. Similarly, we have only tested this method on MicroED data; however, both the implementations for *PHASER* and *PHENIX* refinement are readily able to perform these same calculations on X-ray data, and such data should be compatible with this HAMR method.

### 4.3. Estimated time to completion

As was mentioned previously, the largest bottleneck in this algorithm is the MR process, as when using the default settings of HAMR several thousand conformers must be phased via MR. Because the field of MR applied to small molecules is largely unexplored systematically, it is not easy to predict how long a single MR calculation will take, and during method development a trend was also not easy to deduce. On average, the completion time for all compounds at the highest resolution cutoff is of the order of one to five hours on a personal laptop. This time to completion is generally similar at all resolutions, except at very low resolutions (approximately ≥ 1.5 Å), where multiple HAMR cycles must be performed due to the decreased discriminatory ability of LLG as a metric at these low resolutions, resulting in a noticeably increased time to completion.

## 5. Conclusions

Within this paper, we report a novel high-throughput workflow for structure determination of pharmaceutically relevant molecules using automated MR and MicroED data. This was validated at resolutions ranging from 1.0 to 2.0 Å against three *ab initio* solved structures, which showed good agreement for all HAMR solutions and the respective *ab initio* structures, and publishable *R* factors. Additionally, the method was used to solve a novel macrocycle with pharmaceutical relevance, corilagin, that could not be solved with *ab initio* methods. We predict that this method will see widespread use for even more complex and conformationally flexible molecules than those described here, including important bioactive peptides, toxins and natural products, which typically yield resolutions greater than 1.0 Å. This tool is available for download on request.

## Supplementary Material

Tables S1-S25 and figures S1-S3. DOI: 10.1107/S2052252526002095/vq5007sup1.pdf

Crystal structure: contains datablock(s) 00000001. DOI: 10.1107/S2052252526002095/vq5007sup2.cif

Coordinates for corilagin in PDB format. DOI: 10.1107/S2052252526002095/vq5007sup3.txt

Reflection data for corilagin in MTZ format. DOI: 10.1107/S2052252526002095/vq5007sup4.bin

CCDC reference: 2543412

## Figures and Tables

**Table 1:** Refinement statistics for HAMR solution structures for all validation compounds at varying data resolution limits and RMSD of HAMR solution structures to those previously solved by *ab initio* methods

	Resolution limit (Å)								
	Highest	1.0	1.2	1.4	1.5	1.6	1.8	2.0	2.5
Grazoprevir									
*R*_work_	0.2199	0.2295	0.1962	0.2039	0.1874	0.1693	0.1684	0.1344	0.2881
*R*_free_	0.2506	0.2429	0.2398	0.2241	0.2260	0.1859	0.2066	0.1616	0.5164
RMSD (Å)	0.08	0.30	0.11	0.17	0.29	0.17	0.19	0.29	1.06

Paritaprevir-α									
*R*_work_	0.2360	0.2077	0.1976	0.2015	0.1785	0.2171	0.1683	0.1606	0.3029
*R*_free_	0.2562	0.2353	0.2037	0.2048	0.1901	0.2245	0.1909	0.1730	0.5251
RMSD (Å)	0.06	0.05	0.07	0.10	0.09	0.28	0.12	0.14	2.29

Paritaprevir-β									
*R*_work_	0.2116	0.2047	0.1725	0.1888	0.1749	0.1544	0.1434	0.1401	0.3716
*R*_free_	0.2237	0.2279	0.1955	0.1948	0.1801	0.1843	0.1543	0.1442	0.4699
RMSD (Å)	0.05	0.05	0.06	0.11	0.65	0.65	0.66	0.66	2.92
Adjusted RMSD (Å)	0.05	0.05	0.06	0.11	0.10	0.10	0.13	0.14	2.92

**Table 2:** Experimental details of the crystal structure determination for corilagin

Crystal data	
Chemical formula	C_27_H_22_O_18_·2H_2_O
Crystal system, space group	orthorhombic, *P*2_1_22_1_
Temperature (K)	80
*a*, *b*, *c* (Å)	6.96, 15.89, 24.41
α, β, γ (°)	90, 90, 90
Radiation type, λ (Å)	electrons, 0.0251
Number of crystals	2
Resolution (Å)	1.1

Data collection	
Diffractometer	Talos Arctica TEM
No. of observed reflections	8981
No. of unique reflections	1096
Completeness (%)	84.7
*R*_meas_	0.28
*I*/σ(*I*)	6.42
CC_1/2_ (%)	98.4
	
Refinement	
*R*_work_, *R*_free_	0.2038, 0.2106
No. of reflections in refinement	1026
RMSD_angle_ (°)	2.0
RMSD_bond_ (Å)	0.01
