# Supplementary material for: High-throughput automated molecular replacement for small-molecule MicroED data
Source: IUCrJ. 2026 Apr 10;13(Pt 3):273–81. doi: 10.1107/S2052252526002095 (PMC13134494; doi:10.1107/S2052252526002095)
Supplement: Supplementary file 1 [file m-13-00273-sup1.pdf]

IUCrJ

Volume 13 (2026)

Supporting information for article:

High-throughput automated molecular replacement for small-molecule MicroED data

Adam Thibodeaux, Guanhong Bu, Lael C. Edwards and Emma Rova Danelius

High-Throughput, Automated Molecular Replacement for Small Molecule MicroED Data

Adam Thibodeaux[a], Lael Cardinal[a], Guanhong Bu[a], Emma Rova Danelius[a$]

[a]Department of Chemistry, University California, Riverside

[$]Correspondence to E.R.D emmar@ucr.edu

Supporting Information

Table S1. Comparison of MR statistics for various conformer generators applied to validation compounds.

	Average LLG	Average TFZ
Paritaprevir-α		
CONFORGE	12.79	4.00
EKTDG	12.74	4.00
Paritaprevir-β		
CONFORGE	10.76	3.97
EKTDG	10.71	3.90
Grazoprevir		
CONFORGE	10.65	3.58
EKTDG	9.33	3.36

Table S2. Refinement statistics for *ab initio* structures after MR and refinement in PHENIX for all validation compounds at varying data resolution limits and RMSD of refined *ab initio* solution structures to previously solved by *ab initio* methods.

Resolution Limit	Highest	1.0 Å	1.2 Å	1.4 Å	1.5 Å	1.6 Å	1.8 Å	2.0 Å
Grazoprevir								
R_{work}	0.2187	0.2355	0.2005	0.1891	0.1776	0.1701	0.1722	0.1494
R_{free}	0.2575	0.2166	0.2451	0.1951	0.2689	0.1932	0.2062	0.2466
RMSD (Å)	0.09	0.12	0.11	0.15	0.15	0.16	0.18	0.19
Paritaprevir-α								
R_{work}	0.2348	0.2094	0.2061	0.1962	0.1895	0.1709	0.1638	0.1557
R_{free}	0.2626	0.2385	0.2148	0.1989	0.2161	0.1471	0.3625	0.1825
RMSD (Å)	0.06	0.05	0.08	0.10	0.10	0.10	0.12	0.13
Paritaprevir-β								
R_{work}	0.2212	0.2048	0.1707	0.1794	0.1727	0.1550	0.1388	0.1396
R_{free}	0.2282	0.2263	0.1991	0.1668	0.1341	0.1942	0.1480	0.1262
RMSD (Å)	0.07	0.05	0.06	0.09	0.11	0.10	0.13	0.14

Table S3. Experimental details of the crystal structure determination for grazoprevir at 0.99 Å resolution via HAMR.

Crystal data	
Chemical formula	$C_{38}H_{50}O_9N_6S$
Crystal system, space group	orthorhombic, P 21 21 21
Temperature (K)	80
a, b, c (Å)	6.85, 17.45, 34.51
α, β, γ (°)	90, 90, 90
Radiation type, λ (Å)	electrons, 0.0251
Number of crystals	1
Resolution (Å)	0.99
Data collection	
Diffractometer	Talos Arctica transmission electron microscope
No. of unique reflections	2331
Completeness (%)	92.3
I/σ	3.3
Refinement	
R_{work}, R_{free}	0.2199, 0.2506
No. of reflections in refinement	2214
$RMSD_{angle}$ (°)	4.7
$RMSD_{bond}$ (Å)	0.04

Table S4. Experimental details of the crystal structure determination for grazoprevir at 1.0 Å resolution via HAMR.

Crystal data	
Chemical formula	$C_{38}H_{50}O_9N_6S$
Crystal system, space group	orthorhombic, P 21 21 21
Temperature (K)	80
a, b, c (Å)	6.85, 17.45, 34.51
α, β, γ (°)	90, 90, 90
Radiation type, λ (Å)	electrons, 0.0251
Number of crystals	1
Resolution (Å)	1.0
Data collection	
Diffractometer	Talos Arctica transmission electron microscope
No. of unique reflections	2328
Completeness (%)	92.6
I/σ	3.3
Refinement	
R_{work}, R_{free}	0.2295, 0.2429
No. of reflections in refinement	2211
$RMSD_{angle}$ (°)	4.2
$RMSD_{bond}$ (Å)	0.04

Table S5. Experimental details of the crystal structure determination for grazoprevir at 1.2 Å resolution via HAMR.

Crystal data	
Chemical formula	$C_{38}H_{50}O_9N_6S$
Crystal system, space group	orthorhombic, P 21 21 21
Temperature (K)	80
a, b, c (Å)	6.85, 17.45, 34.51
α, β, γ (°)	90, 90, 90
Radiation type, λ (Å)	electrons, 0.0251
Number of crystals	1
Resolution (Å)	1.2
Data collection	
Diffractometer	Talos Arctica transmission electron microscope
No. of unique reflections	1386
Completeness (%)	92.1
I/σ	4.8
Refinement	
R_{work}, R_{free}	0.1962, 0.2398
No. of reflections in refinement	1316
$RMSD_{angle}$ (°)	3.7
$RMSD_{bond}$ (Å)	0.03

Table S6. Experimental details of the crystal structure determination for grazoprevir at 1.4 Å resolution via HAMR.

Crystal data	
Chemical formula	$C_{38}H_{50}O_9N_6S$
Crystal system, space group	orthorhombic, P 21 21 21
Temperature (K)	80
a, b, c (Å)	6.85, 17.45, 34.51
α, β, γ (°)	90, 90, 90
Radiation type, λ (Å)	electrons, 0.0251
Number of crystals	1
Resolution (Å)	1.4
Data collection	
Diffractometer	Talos Arctica transmission electron microscope
No. of unique reflections	879
Completeness (%)	92.1
I/σ	6.1
Refinement	
R_{work}, R_{free}	0.2039, 0.2241
No. of reflections in refinement	835
$RMSD_{angle}$ (°)	2.4
$RMSD_{bond}$ (Å)	0.01

Table S7. Experimental details of the crystal structure determination for grazoprevir at 1.5 Å resolution via HAMR.

Crystal data	
Chemical formula	$C_{38}H_{50}O_9N_6S$
Crystal system, space group	orthorhombic, P 21 21 21
Temperature (K)	80
a, b, c (Å)	6.85, 17.45, 34.51
α, β, γ (°)	90, 90, 90
Radiation type, λ (Å)	electrons, 0.0251
Number of crystals	1
Resolution (Å)	1.5
Data collection	
Diffractometer	Talos Arctica transmission electron microscope
No. of unique reflections	730
Completeness (%)	90.9
I/σ	6.7
Refinement	
R_{work}, R_{free}	0.1874, 0.2260
No. of reflections in refinement	693
$RMSD_{angle}$ (°)	2.9
$RMSD_{bond}$ (Å)	0.02

Table S8. Experimental details of the crystal structure determination for grazoprevir at 1.6 Å resolution via HAMR.

Crystal data	
Chemical formula	$C_{38}H_{50}O_9N_6S$
Crystal system, space group	orthorhombic, P 21 21 21
Temperature (K)	80
a, b, c (Å)	6.85, 17.45, 34.51
α, β, γ (°)	90, 90, 90
Radiation type, λ (Å)	electrons, 0.0251
Number of crystals	1
Resolution (Å)	1.6
Data collection	
Diffractometer	Talos Arctica transmission electron microscope
No. of unique reflections	603
Completeness (%)	90.9
I/σ	7.2
Refinement	
R_{work}, R_{free}	0.1693, 0.1859
No. of reflections in refinement	572
$RMSD_{angle}$ (°)	2.8
$RMSD_{bond}$ (Å)	0.02

Table S9. Experimental details of the crystal structure determination for grazoprevir at 1.8 Å resolution via HAMR.

Crystal data	
Chemical formula	$C_{38}H_{50}O_9N_6S$
Crystal system, space group	orthorhombic, P 21 21 21
Temperature (K)	80
a, b, c (Å)	6.85, 17.45, 34.51
α, β, γ (°)	90, 90, 90
Radiation type, λ (Å)	electrons, 0.0251
Number of crystals	1
Resolution (Å)	1.8
Data collection	
Diffractometer	Talos Arctica transmission electron microscope
No. of unique reflections	430
Completeness (%)	90.0
I/σ	8.0
Refinement	
R_{work}, R_{free}	0.1684, 0.2066
No. of reflections in refinement	408
$RMSD_{angle}$ (°)	2.3
$RMSD_{bond}$ (Å)	0.01

Table S9. Experimental details of the crystal structure determination for grazoprevir at 2.0 Å resolution via HAMR.

Crystal data	
Chemical formula	$C_{38}H_{50}O_9N_6S$
Crystal system, space group	orthorhombic, P 21 21 21
Temperature (K)	80
a, b, c (Å)	6.85, 17.45, 34.51
α, β, γ (°)	90, 90, 90
Radiation type, λ (Å)	electrons, 0.0251
Number of crystals	1
Resolution (Å)	2.0
Data collection	
Diffractometer	Talos Arctica transmission electron microscope
No. of unique reflections	326
Completeness (%)	90.0
I/σ	8.6
Refinement	
R_{work}, R_{free}	0.1344, 0.1616
No. of reflections in refinement	309
$RMSD_{angle}$ (°)	2.9
$RMSD_{bond}$ (Å)	0.02

Table S10. Experimental details of the crystal structure determination for paritaprevir-α at 0.85 Å resolution via HAMR.

Crystal data	
Chemical formula	$C_{40}H_{43}O_7N_7S$
Crystal system, space group	orthorhombic, P 21 21 21
Temperature (K)	80
a, b, c (Å)	5.09, 15.61, 50.78
α, β, γ (°)	90, 90, 90
Radiation type, λ (Å)	electrons, 0.0251
Number of crystals	1
Resolution (Å)	0.85
Data collection	
Diffractometer	Talos Arctica transmission electron microscope
No. of unique reflections	3604
Completeness (%)	89.9
I/σ	2.8
Refinement	
R_{work}, R_{free}	0.2360, 0.2562
No. of reflections in refinement	3423
$RMSD_{angle}$ (°)	3.5
$RMSD_{bond}$ (Å)	0.02

Table S11. Experimental details of the crystal structure determination for paritaprevir-α at 1.0 Å resolution via HAMR.

Crystal data	
Chemical formula	$C_{40}H_{43}O_7N_7S$
Crystal system, space group	orthorhombic, P 21 21 21
Temperature (K)	80
a, b, c (Å)	5.09, 15.61, 50.78
α, β, γ (°)	90, 90, 90
Radiation type, λ (Å)	electrons, 0.0251
Number of crystals	1
Resolution (Å)	1.0
Data collection	
Diffractometer	Talos Arctica transmission electron microscope
No. of unique reflections	2226
Completeness (%)	88.1
I/σ	3.9
Refinement	
R_{work}, R_{free}	0.2077, 0.2353
No. of reflections in refinement	2114
$RMSD_{angle}$ (°)	4.1
$RMSD_{bond}$ (Å)	0.03

Table S12. Experimental details of the crystal structure determination for paritaprevir-α at 1.2 Å resolution via HAMR.

Crystal data	
Chemical formula	$C_{40}H_{43}O_7N_7S$
Crystal system, space group	orthorhombic, P 21 21 21
Temperature (K)	80
a, b, c (Å)	5.09, 15.61, 50.78
α, β, γ (°)	90, 90, 90
Radiation type, λ (Å)	electrons, 0.0251
Number of crystals	1
Resolution (Å)	1.2
Data collection	
Diffractometer	Talos Arctica transmission electron microscope
No. of unique reflections	1291
Completeness (%)	84.9
I/σ	4.9
Refinement	
R_{work}, R_{free}	0.1976, 0.2037
No. of reflections in refinement	1226
$RMSD_{angle}$ (°)	3.5
$RMSD_{bond}$ (Å)	0.02

Table S13. Experimental details of the crystal structure determination for paritaprevir-α at 1.4 Å resolution via HAMR.

Crystal data	
Chemical formula	$C_{40}H_{43}O_7N_7S$
Crystal system, space group	orthorhombic, P 21 21 21
Temperature (K)	80
a, b, c (Å)	5.09, 15.61, 50.78
α, β, γ (°)	90, 90, 90
Radiation type, λ (Å)	electrons, 0.0251
Number of crystals	1
Resolution (Å)	1.4
Data collection	
Diffractometer	Talos Arctica transmission electron microscope
No. of unique reflections	809
Completeness (%)	81.8
I/σ	5.5
Refinement	
R_{work}, R_{free}	0.2015, 0.2048
No. of reflections in refinement	768
$RMSD_{angle}$ (°)	2.4
$RMSD_{bond}$ (Å)	0.01

Table S14. Experimental details of the crystal structure determination for paritaprevir-α at 1.5 Å resolution via HAMR.

Crystal data	
Chemical formula	$C_{40}H_{43}O_7N_7S$
Crystal system, space group	orthorhombic, P 21 21 21
Temperature (K)	80
a, b, c (Å)	5.09, 15.61, 50.78
α, β, γ (°)	90, 90, 90
Radiation type, λ (Å)	electrons, 0.0251
Number of crystals	1
Resolution (Å)	1.5
Data collection	
Diffractometer	Talos Arctica transmission electron microscope
No. of unique reflections	658
Completeness (%)	80.9
I/σ	5.8
Refinement	
R_{work}, R_{free}	0.1785, 0.1901
No. of reflections in refinement	625
$RMSD_{angle}$ (°)	3.1
$RMSD_{bond}$ (Å)	0.01

Table S15. Experimental details of the crystal structure determination for paritaprevir-α at 1.6 Å resolution via HAMR.

Crystal data	
Chemical formula	$C_{40}H_{43}O_7N_7S$
Crystal system, space group	orthorhombic, P 21 21 21
Temperature (K)	80
a, b, c (Å)	5.09, 15.61, 50.78
α, β, γ (°)	90, 90, 90
Radiation type, λ (Å)	electrons, 0.0251
Number of crystals	1
Resolution (Å)	1.6
Data collection	
Diffractometer	Talos Arctica transmission electron microscope
No. of unique reflections	541
Completeness (%)	79.7
I/σ	6.0
Refinement	
R_{work}, R_{free}	0.2171, 0.2245
No. of reflections in refinement	514
$RMSD_{angle}$ (°)	3.1
$RMSD_{bond}$ (Å)	0.02

Table S16. Experimental details of the crystal structure determination for paritaprevir-α at 1.8 Å resolution via HAMR.

Crystal data	
Chemical formula	$C_{40}H_{43}O_7N_7S$
Crystal system, space group	orthorhombic, P 21 21 21
Temperature (K)	80
a, b, c (Å)	5.09, 15.61, 50.78
α, β, γ (°)	90, 90, 90
Radiation type, λ (Å)	electrons, 0.0251
Number of crystals	1
Resolution (Å)	1.8
Data collection	
Diffractometer	Talos Arctica transmission electron microscope
No. of unique reflections	374
Completeness (%)	77.0
I/σ	6.5
Refinement	
R_{work}, R_{free}	0.1683, 0.1909
No. of reflections in refinement	336
$RMSD_{angle}$ (°)	3.0
$RMSD_{bond}$ (Å)	0.02

Table S17. Experimental details of the crystal structure determination for paritaprevir-α at 2.0 Å resolution via HAMR.

Crystal data	
Chemical formula	$C_{40}H_{43}O_7N_7S$
Crystal system, space group	orthorhombic, P 21 21 21
Temperature (K)	80
a, b, c (Å)	5.09, 15.61, 50.78
α, β, γ (°)	90, 90, 90
Radiation type, λ (Å)	electrons, 0.0251
Number of crystals	1
Resolution (Å)	2.0
Data collection	
Diffractometer	Talos Arctica transmission electron microscope
No. of unique reflections	282
Completeness (%)	76.8
I/σ	6.7
Refinement	
R_{work}, R_{free}	0.1606, 0.1730
No. of reflections in refinement	267
$RMSD_{angle}$ (°)	2.8
$RMSD_{bond}$ (Å)	0.01

Table S18. Experimental details of the crystal structure determination for paritaprevir-β at 0.95 Å resolution via HAMR.

Crystal data	
Chemical formula	$C_{40}H_{43}O_7N_7S$
Crystal system, space group	orthorhombic, P 21 21 21
Temperature (K)	80
a, b, c (Å)	10.56, 12.32, 31.73
α, β, γ (°)	90, 90, 90
Radiation type, λ (Å)	electrons, 0.0251
Number of crystals	1
Resolution (Å)	0.95
Data collection	
Diffractometer	Talos Arctica transmission electron microscope
No. of unique reflections	2824
Completeness (%)	98.5
I/σ	2.3
Refinement	
R_{work}, R_{free}	0.2116, 0.2237
No. of reflections in refinement	2682
$RMSD_{angle}$ (°)	4.4
$RMSD_{bond}$ (Å)	0.03

Table S19. Experimental details of the crystal structure determination for paritaprevir-β at 1.0 Å resolution via HAMR.

Crystal data	
Chemical formula	$C_{40}H_{43}O_7N_7S$
Crystal system, space group	orthorhombic, P 21 21 21
Temperature (K)	80
a, b, c (Å)	10.56, 12.32, 31.73
α, β, γ (°)	90, 90, 90
Radiation type, λ (Å)	electrons, 0.0251
Number of crystals	1
Resolution (Å)	1.0
Data collection	
Diffractometer	Talos Arctica transmission electron microscope
No. of unique reflections	2482
Completeness (%)	99.8
I/σ	2.5
Refinement	
R_{work}, R_{free}	0.2047, 0.2279
No. of reflections in refinement	2357
$RMSD_{angle}$ (°)	4.5
$RMSD_{bond}$ (Å)	0.03

Table S20. Experimental details of the crystal structure determination for paritaprevir-β at 1.2 Å resolution via HAMR.

Crystal data	
Chemical formula	$C_{40}H_{43}O_7N_7S$
Crystal system, space group	orthorhombic, P 21 21 21
Temperature (K)	80
a, b, c (Å)	10.56, 12.32, 31.73
α, β, γ (°)	90, 90, 90
Radiation type, λ (Å)	electrons, 0.0251
Number of crystals	1
Resolution (Å)	1.2
Data collection	
Diffractometer	Talos Arctica transmission electron microscope
No. of unique reflections	1472
Completeness (%)	99.5
I/σ	3.4
Refinement	
R_{work}, R_{free}	0.1725, 0.1955
No. of reflections in refinement	1398
$RMSD_{angle}$ (°)	4.5
$RMSD_{bond}$ (Å)	0.04

Table S21. Experimental details of the crystal structure determination for paritaprevir-β at 1.4 Å resolution via HAMR.

Crystal data	
Chemical formula	$C_{40}H_{43}O_7N_7S$
Crystal system, space group	orthorhombic, P 21 21 21
Temperature (K)	80
a, b, c (Å)	10.56, 12.32, 31.73
α, β, γ (°)	90, 90, 90
Radiation type, λ (Å)	electrons, 0.0251
Number of crystals	1
Resolution (Å)	1.4
Data collection	
Diffractometer	Talos Arctica transmission electron microscope
No. of unique reflections	950
Completeness (%)	99.4
I/σ	4.0
Refinement	
R_{work}, R_{free}	0.1749, 0.1801
No. of reflections in refinement	902
$RMSD_{angle}$ (°)	2.9
$RMSD_{bond}$ (Å)	0.01

Table S22. Experimental details of the crystal structure determination for paritaprevir-β at 1.5 Å resolution via HAMR.

Crystal data	
Chemical formula	$C_{40}H_{43}O_7N_7S$
Crystal system, space group	orthorhombic, P 21 21 21
Temperature (K)	80
a, b, c (Å)	10.56, 12.32, 31.73
α, β, γ (°)	90, 90, 90
Radiation type, λ (Å)	electrons, 0.0251
Number of crystals	1
Resolution (Å)	1.5
Data collection	
Diffractometer	Talos Arctica transmission electron microscope
No. of unique reflections	772
Completeness (%)	99.4
I/σ	4.3
Refinement	
R_{work}, R_{free}	0.1544, 0.1843
No. of reflections in refinement	733
$RMSD_{angle}$ (°)	3.5
$RMSD_{bond}$ (Å)	0.01

Table S23. Experimental details of the crystal structure determination for paritaprevir-β at 1.6 Å resolution via HAMR.

Crystal data	
Chemical formula	$C_{40}H_{43}O_7N_7S$
Crystal system, space group	orthorhombic, P 21 21 21
Temperature (K)	80
a, b, c (Å)	10.56, 12.32, 31.73
α, β, γ (°)	90, 90, 90
Radiation type, λ (Å)	electrons, 0.0251
Number of crystals	1
Resolution (Å)	1.6
Data collection	
Diffractometer	Talos Arctica transmission electron microscope
No. of unique reflections	643
Completeness (%)	99.2
I/σ	4.5
Refinement	
R_{work}, R_{free}	0.1888, 0.1948
No. of reflections in refinement	610
$RMSD_{angle}$ (°)	3.5
$RMSD_{bond}$ (Å)	0.01

Table S24. Experimental details of the crystal structure determination for paritaprevir-β at 1.8 Å resolution via HAMR.

Crystal data	
Chemical formula	$C_{40}H_{43}O_7N_7S$
Crystal system, space group	orthorhombic, P 21 21 21
Temperature (K)	80
a, b, c (Å)	10.56, 12.32, 31.73
α, β, γ (°)	90, 90, 90
Radiation type, λ (Å)	electrons, 0.0251
Number of crystals	1
Resolution (Å)	1.8
Data collection	
Diffractometer	Talos Arctica transmission electron microscope
No. of unique reflections	451
Completeness (%)	98.2
I/σ	4.9
Refinement	
R_{work}, R_{free}	0.1434, 0.1543
No. of reflections in refinement	428
$RMSD_{angle}$ (°)	2.8
$RMSD_{bond}$ (Å)	0.01

Table S25. Experimental details of the crystal structure determination for paritaprevir-β at 2.0 Å resolution via HAMR.

Crystal data	
Chemical formula	$C_{40}H_{43}O_7N_7S$
Crystal system, space group	orthorhombic, P 21 21 21
Temperature (K)	80
a, b, c (Å)	10.56, 12.32, 31.73
α, β, γ (°)	90, 90, 90
Radiation type, λ (Å)	electrons, 0.0251
Number of crystals	1
Resolution (Å)	2.0
Data collection	
Diffractometer	Talos Arctica transmission electron microscope
No. of unique reflections	342
Completeness (%)	98.6
I/σ	4.9
Refinement	
R_{work}, R_{free}	0.1401, 0.1442
No. of reflections in refinement	324
$RMSD_{angle}$ (°)	2.7
$RMSD_{bond}$ (Å)	0.01

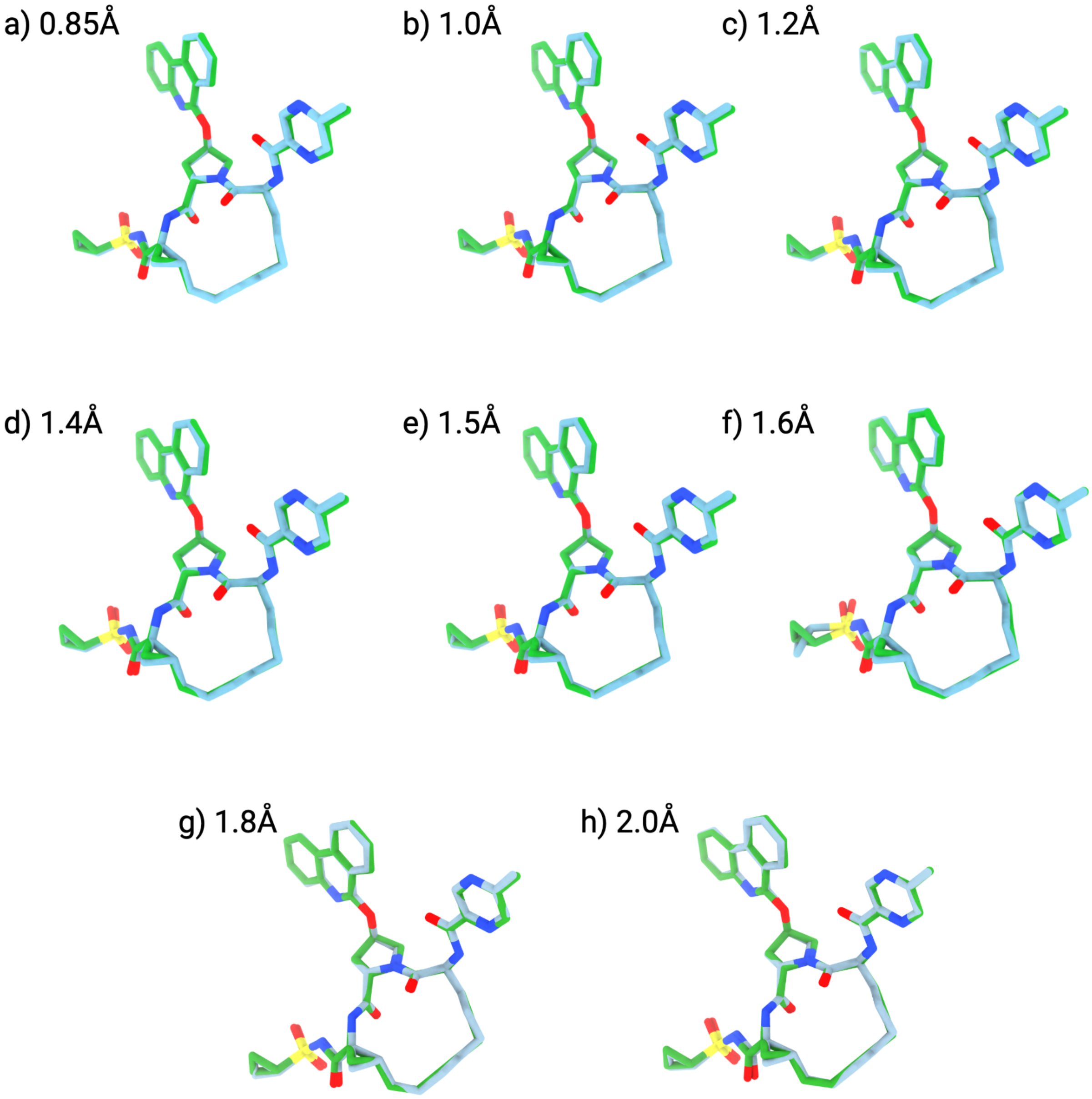

Figure S1. Comparison of structure solved with *ab initio* methods (green) at highest resolution to HAMR solutions (light blue) at various data resolutions for paritaparevir-α.

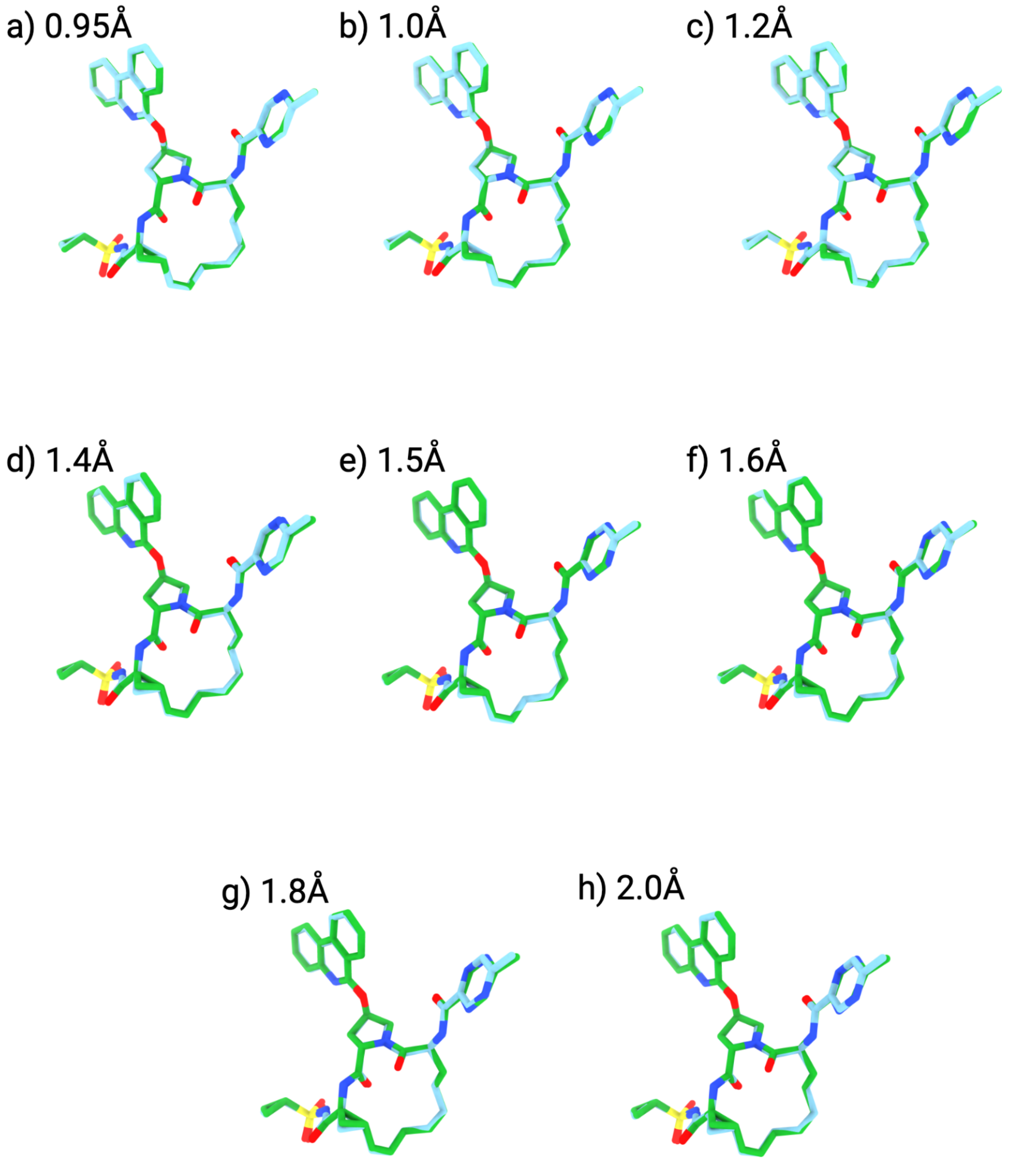

Figure S2. Comparison of structure solved with *ab initio* methods (green) at highest resolution to HAMR solutions (light blue) at various data resolutions for paritaparevir-β.

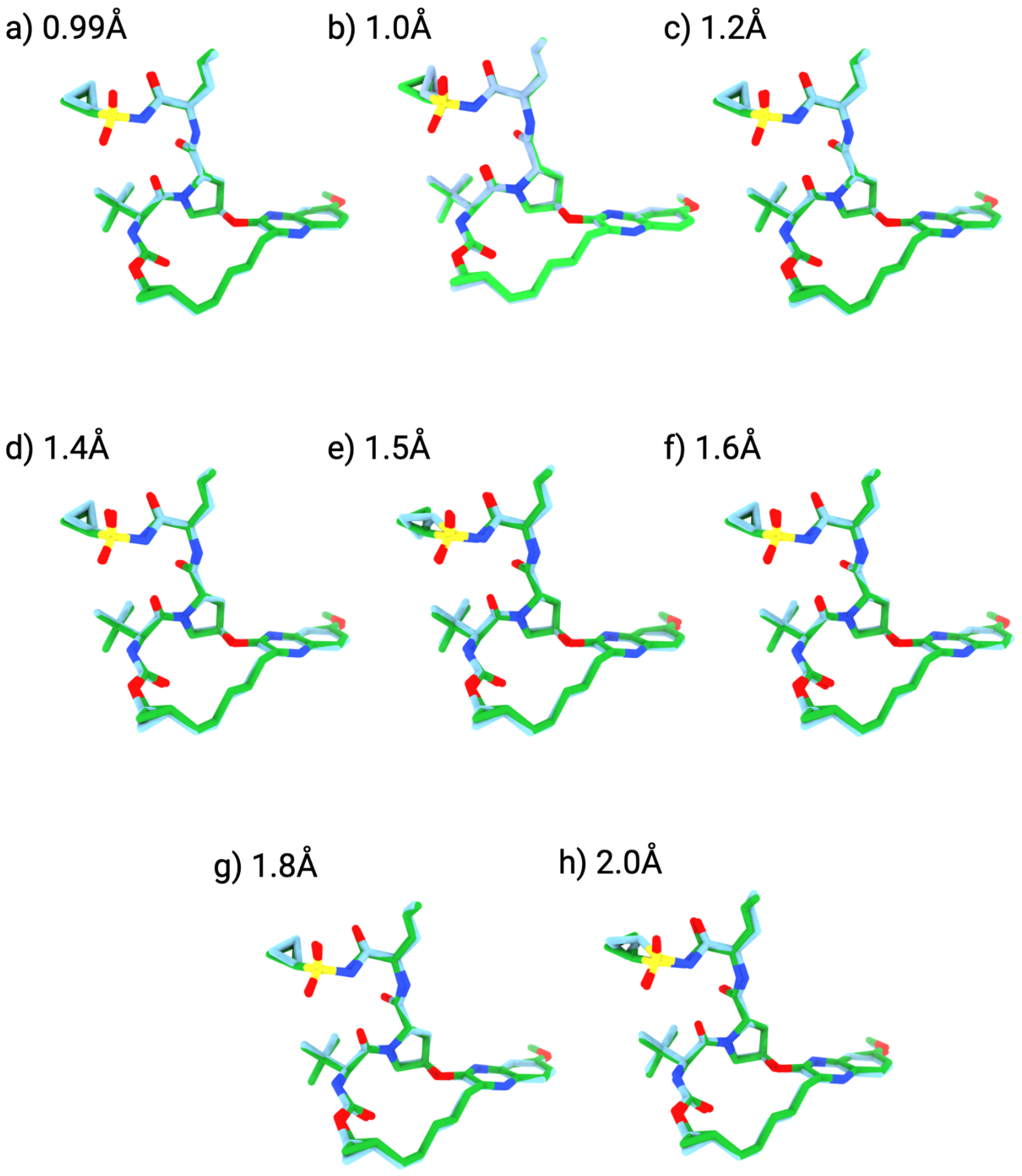

Figure S3. Comparison of structure solved with *ab initio* methods (green) at highest resolution to HAMR solutions (light blue) at various data resolutions for grazoprevir.
